# Work at Heights Training: Conventional Approach with and Without Immersive Virtual Reality Study Protocol

**DOI:** 10.3390/mps9020055

**Published:** 2026-04-01

**Authors:** Diana Guerrero-Jaramillo, Ricardo de la Caridad Montero, Oscar Campo

**Affiliations:** 1 Facultad de Ingeniería, Universidad del Valle, Cali 760030, Colombia; 2 Facultad de Ingeniería y Ciencias Básicas, Universidad Autónoma de Occidente, Cali 760030, Colombia; rcmontero@uao.edu.co (R.d.l.C.M.); oicampo@uao.edu.co (O.C.)

**Keywords:** virtual reality, occupational health, outcome evaluation, training

## Abstract

Background: Work at heights is a high-risk occupational activity, with falls being a leading cause of fatal accidents in construction and industrial maintenance. Conventional safety training often does not fully prepare workers for real-world hazards. Immersive virtual reality (IVR) has emerged as a promising training tool, providing controlled and realistic simulations of hazardous scenarios. This hypothesis-generating pilot study evaluates the feasibility and effectiveness of IVR in enhancing practical skills, safety perception, and physiological responses during work-at-height training. Methods: This controlled trial will recruit first-time trainees from the National Learning Service (SENA) of Colombia. Participants will be assigned to an intervention group, receiving IVR training before field-based practical sessions, or a control group, receiving standard theoretical instruction. Outcomes include practical skill acquisition, ergonomic risk, cognitive performance, and physiological responses, including heart rate variability measured with validated devices. Assessments will be performed using standardized tools, and data will be analyzed with repeated-measures ANOVA and regression models to compare groups. Conclusions: By integrating practical, cognitive, ergonomic, and physiological measures, this study will provide evidence on whether IVR improves the effectiveness of work-at-height training beyond conventional methods. Findings may inform future strategies to enhance occupational safety training in high-risk work environments.

## 1. Introduction

Work at heights is one of the occupational activities associated with the highest risk of serious work-related injuries and fatalities, with falls representing one of the leading causes of death in sectors such as construction, electricity, and industrial maintenance [1]. Recognizing this risk, the International Labour Organization (ILO), through Conventions 155 and 167, establishes guidelines to ensure safety in work-at-height activities, regulating key aspects such as fall prevention, mandatory worker training, and the appropriate use of personal protective equipment. These guidelines include the implementation of safety systems (e.g., guardrails, safety nets, and harnesses), systematic risk assessment prior to task execution, supervision and equipment maintenance, and shared responsibility between employers and workers to comply with safety regulations.

Despite these regulatory efforts, work-at-height activities continue to account for a substantial proportion of occupational fatalities worldwide. According to the ILO, approximately 2.78 million workers die each year globally due to severe occupational injuries, a significant proportion of which are caused by falls [2]. In the United States, data from the Bureau of Labor Statistics indicate that falls, slips, and trips accounted for a substantial number of deaths in the construction sector, with 370 fatalities reported in construction and extraction occupations in 2021 alone [3]. Evidence from China further highlights the magnitude of this risk, with 1919 incidents of falls from heights reported between 2017 and 2021, accounting for 53% of housing and municipal engineering safety accidents, underscoring the persistent global burden of fall-related events in construction contexts [3,4]. In Colombia, during the first quarter of 2024, 16,850 occupational accidents were reported in the construction sector, including 90 fatalities, of which approximately 20% were related to work at heights [5]. Beyond the irreversible human cost, these incidents generate significant economic losses and hinder sustainable industrial development.

These data underscore the need to implement innovative strategies to improve worker training and reduce the risks associated with work-at-height activities. In this context, there has been growing interest in the use of virtual reality (VR) as a tool for occupational safety training, given its capacity to simulate hazardous situations in controlled environments while capturing users’ attention and engagement.

VR is a technology that creates immersive, computer-generated environments that simulate real or imagined settings, allowing users to interact with virtual objects in real time. It is widely used across sectors such as education, training, health, and entertainment to enhance engagement and experiential learning [6].

Several studies have demonstrated the effectiveness of VR for hazard identification and risk recognition. Perlman et al. [7], for example, compared hazard recognition capabilities between participants trained using traditional images and documents and those trained using VR, reporting superior risk identification among participants exposed to VR-based training.

Comparative studies have also evaluated different VR-based training modalities. Eiris et al. [8] compared a 360-degree panoramic visualization of a construction site with a static virtual reality environment and found that the static VR condition achieved a higher hazard identification index. Jeelani et al. [9] developed an interoperable system combining VR and stereoscopic panoramic environments, which improved hazard identification but at the cost of increased system complexity and development expenses. Although VR shows considerable potential, its widespread adoption remains limited due to the time and cost required for content development. To address these barriers, some studies have incorporated gaming technologies with interactive risk scenarios, while others have explored reinforcement learning approaches combined with VR and motion tracking to improve safety-related behaviors [10,11].

Additional approaches have included the use of Building Information Modeling (BIM) 4D integrated with game engines for safety training, as well as the incorporation of audio and video instructions to enhance virtual environments [12,13]. Pooladvand et al. developed a crane simulator to improve user interaction during training processes [14]. In Colombia, a textbook from the Universidad Autónoma de Occidente highlighted the potential of VR for work-at-height training, emphasizing its capacity to create active, problem-based learning environments that connect theoretical concepts with real-world scenarios [15].

Previous international studies have demonstrated the potential of virtual reality for safety training and acrophobia exposure, showing improvements in safety awareness, operational skills, and behavioral responses in simulated high-risk environments [11,16]. In addition, VR-based proprioceptive and exposure interventions have shown benefits in balance control and fear-of-height responses [17]. However, much of this research has relied on desktop-based or semi-immersive systems, emphasized cognitive or behavioral outcomes, and rarely incorporated objective physiological indicators. In the national context, no published studies have been identified that employ fully immersive VR for occupational safety training combined with multidimensional evaluation frameworks.

The present study seeks to advance this line of research through the implementation of immersive virtual reality rather than conventional desktop-based VR. More realistic scenarios will be developed within a serious game framework, new measurement variables will be incorporated, and the experimental methodology will be refined to optimize the evaluation of the impact of immersive VR on work-at-height training outcomes.

Recent studies have also emphasized the importance of collecting user data within virtual environments to enhance training effectiveness. Jacobsen et al. [18] proposed a framework for collecting user data across hazardous scenarios and automating VR-based evaluations. Other studies have tracked users’ spatial positions within mobile VR environments and integrated these data into BIM 360 software (Autodesk, San Francisco, CA, USA) for detailed analyses of workers’ spatial awareness.

Despite its potential, research on VR-based safety training in high-risk sectors such as construction remains limited. Team-based work-at-height activities pose additional challenges related to communication and coordination, which can complicate the implementation of VR technologies. Nevertheless, existing evidence highlights the potential of VR to improve safety behaviors and worker engagement in hazardous environments [19].

Moreover, analyzing the specific tasks performed during work-at-height activities enables the design of VR environments that more accurately reflect real-world conditions, including structural characteristics, tools used, and task-specific risks. In this context, immersive virtual reality emerges as a promising tool for occupational safety training, as it allows the recreation of realistic work scenarios in controlled environments where workers can practice safety procedures without exposure to real hazards [13]. In addition to providing a safe and practical training experience, VR has been shown to outperform traditional training methods by improving practical skills, executive memory, and decision-making in critical situations [20,21], although such effects may be influenced by differences in training exposure.

Incorporating biosignal measurements during training may provide objective indicators of training effectiveness. Parameters such as heart rate and heart rate variability offer quantitative information regarding workers’ physical and emotional states, allowing the identification of stress, anxiety, or fatigue levels and facilitating individualized training adaptations [22,23]. By integrating immersive virtual reality with physiological and emotional response assessment, this hypothesis-generating pilot study aims to evaluate the feasibility and effectiveness of conventional training complemented with immersive VR for work-at-height activities, analyzing its impact on practical skill acquisition, safety perception, and physiological responses. This integrated approach seeks not only to reduce accidents and associated costs but also to generate new knowledge regarding the influence of physiological and emotional responses on performance in high-risk occupational activities [24].

Beyond addressing these objectives, the present protocol introduces several methodological and technological innovations that extend prior research in this field. It employs fully immersive virtual reality with embodied interaction, enabling users to perform safety-critical tasks rather than merely observe hazards in desktop-based environments. In addition, it adopts a multidimensional evaluation framework integrating practical performance, ergonomic risk, cognitive function, and physiological responses, allowing a comprehensive assessment of training effectiveness. The incorporation of heart rate variability provides objective insight into autonomic and stress responses during training, an aspect rarely explored in work-at-height simulations. Finally, the virtual scenarios were developed based on real task analysis to enhance ecological validity and support behavioral transfer to real-world settings.

## 2. Experimental Design

This study is designed as a pilot-controlled trial with outcome assessor blinding, aimed at evaluating the effects and safety of an immersive virtual reality-based training intervention for work-at-height activities. Participants will be randomly assigned to one of two study groups: an intervention group receiving immersive virtual reality-based work-at-height training prior to practical training in a real-world setting, and a control group receiving the standard training currently implemented, consisting of theoretical instruction without immersive virtual reality. This design allows for the comparison of outcomes between groups to assess differences associated with the addition of immersive virtual reality to conventional training.

**Figure 1:**
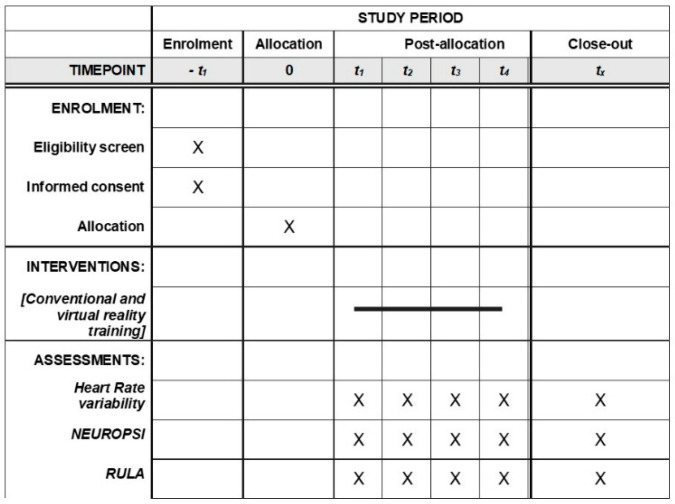
Screening and consent occur before allocation (t − 1). Participants receive conventional or VR training at allocation (t0). Heart rate variability, NEUROPSI, and RULA are measured at multiple post-allocation timepoints (t1–t4) and at study end (t_x_). X indicates the time point at which each assessment is performed. The horizontal line represents the duration of the intervention period. Timepoints are presented relative to allocation (e.g., −1 indicates pre-allocation). The total estimated duration of the experimental session at t0 is approximately 30–40 min.

A subset corresponding to 50% of participants from each study group will be randomly selected for a three-month follow-up to assess the persistence of training-related outcomes, including skill retention and behavioral transfer. The inclusion and exclusion criteria for follow-up are identical to those defined for the main study population. This follow-up was designed to explore skill retention and behavioral transfer over time, providing preliminary longitudinal evidence to inform the design of future fully powered long-term trials. All participants will be individuals performing activities within the construction sector. The trial will be conducted in accordance with the Consolidated Standards of Reporting Trials (CONSORT) guidelines [25]. In addition, the study protocol was developed following the recommendations of the SPIRIT guidelines (Figure S1 and File S1) [26]. The study was registered at https://ClinicalTrials.gov (NCT06728566) on 24 October 2024, prior to the initiation of the clinical trial (Figure 1).

### 2.1. Participants, Eligibility Criteria, and Recruitment

Eligible participants will be healthy adults of any sex, aged between 18 and 65 years, who meet the regulatory requirements for work-at-height activities. All participants must fulfill the established inclusion criteria to undergo immersive virtual reality-based training.

Participants will be recruited from apprentices enrolled at the National Learning Service (Servicio Nacional de Aprendizaje, SENA) in Colombia who are undertaking work-at-height training for the first time. Prior to enrollment, all participants will provide written informed consent, and compliance with the mandatory occupational medical examinations required by current regulations will be verified.

Individuals with prior experience in work-at-height activities will be excluded, as well as those presenting medical or health conditions that could prevent safe participation in immersive virtual reality training or in work-at-height practical training.

#### 2.1.1. Recruitment

Participant recruitment will be conducted in collaboration with SENA by identifying eligible candidates who meet the predefined inclusion and exclusion criteria. Potential participants will be informed about the study objectives, procedures, and potential risks, and written informed consent will be obtained before the initiation of any study-related activities. Selected participants will receive electronic communication detailing the date, time, and location of the study procedures.

#### 2.1.2. Study Status and Timeline

At the time of writing, no results have been generated from this study. Participant recruitment began on 01/08/2025 and is currently ongoing, with completion expected by June 2026. Data collection and analysis will be conducted following the completion of participant recruitment. Final analyses and reporting of results are anticipated during the first half of 2027.

## 3. Procedure

### 3.1. Interventions

Participants allocated to the intervention group will receive work-at-height training complemented with immersive virtual reality, whereas participants in the control group will receive conventional training only. For both groups, the standard training program will consist of six hours of theoretical instruction and four hours of practical training. In addition to this standard training, the intervention group will complete a 30 min immersive virtual reality session focused on work-at-height activities. Exposure to training will be standardized and recorded across groups (total training time in minutes), and will be considered in the analysis to ensure comparability and reproducibility.

The conventional training provided to both groups will include a standardized sequence of safety procedures and equipment use. Participants will be instructed to inspect the harness for structural damage, correctly don the harness in a vest-like configuration, and adjust shoulder and leg straps to ensure comfort and safety. A final inspection will be performed to verify correct positioning of the D-rings and secure fastening of all buckles. Helmet inspection and proper adjustment will be conducted to ensure adequate fit. Lanyards will be selected based on appropriate conditions and compatibility, ensuring correct connection and adequate tension without excessive load. Certified carabiners with functional locking mechanisms will be used and connected to appropriate anchorage points. Lifelines and energy absorbers will be correctly installed and tightened, and their condition will be verified prior to use. During ascent and descent maneuvers, participants will be instructed to maintain three points of contact at all times and to secure tools to prevent falls.

For the immersive virtual reality intervention, a dedicated technological ecosystem will be implemented to provide an interactive and realistic training experience. The Meta Quest 2 device will be used (Meta Platforms Technologies, LLC, Menlo Park, CA, USA), featuring a resolution of 1832 × 1920 pixels per eye, an adaptive refresh rate between 72 and 90 Hz, and inside-out tracking with six degrees of freedom (6DoF). The virtual environment will be developed using Unity 2022.x, integrating the Meta Interaction SDK to optimize interaction, tracking, and rendering. Additionally, an Arduino ESP32 system connected to two potentiometers mounted on a fiberglass platform will be incorporated to detect step pressure changes. Analog signals will be processed to identify user steps in real time. To reduce implementation-related bias, the VR system will undergo prior technical validation and pilot testing to ensure usability, stability, and a consistent user experience, minimizing the influence of system performance on study outcomes.

Virtual reality scenarios will be designed based on ergonomic, safety, and realism criteria to ensure ecological validity. Scenario 1 (Personal Protective Equipment Inspection) will require participants to visually and tactually inspect harnesses, helmets, lanyards, and carabiners, receiving visual and auditory feedback on detected faults. Scenario 2 (Ascent on a Metallic Structure) will train correct hooking and unhooking techniques, incorporating gravity simulation, natural movement detection, and verification of anchorage connections with visual, vibratory, and error alerts. Scenario 3 (Safe Positioning and Descent) will expose participants to simulated environmental conditions, realistic tool manipulation, and anchorage transitions, with performance penalties applied for safety errors to promote progressive learning.

To enhance immersion and safety, system performance will be optimized to maintain stable frame rates and minimize motion sickness. An intuitive user interface with natural controls and haptic feedback will be implemented. Performance metrics, including task successes and errors, will be recorded automatically to provide structured feedback and support learning progression.

### 3.2. Measurements

#### 3.2.1. Practical Skills for Work at Heights

Practical skills will be evaluated using a standardized and structured checklist routinely employed by certified work-at-height instructors from the National Learning Service of Colombia. The assessment will encompass all predefined safety-critical domains required for safe work-at-height performance, including equipment use, anchorage management, mobility at height, and emergency procedures. Each domain will be evaluated independently and rated using a dichotomous pass/fail system, with a “pass” score assigned only when all domain-specific safety criteria are fully met. Assessments will be conducted immediately after completion of the practical training phase

#### 3.2.2. Cardiac Autonomic Response

Cardiac autonomic function will be assessed through heart rate variability (HRV) analysis derived from beat-to-beat heart rate recordings obtained using a Polar H10 heart rate sensor (Polar Electro Oy, Kempele, Finland). The Polar H10 is a chest-strap device equipped with integrated electrodes capable of recording RR intervals with high temporal resolution and has been widely used in research settings for the assessment of autonomic cardiac modulation during resting conditions, physical activity, and exposure to stressors. RR intervals will be obtained under three standardized conditions: (1) a 5 min seated resting baseline prior to training; (2) task execution, including ascent and descent maneuvers and/or immersive virtual reality exposure, depending on group allocation; and (3) a 5 min seated recovery period after task completion.

The data will be recorded continuously during baseline, training, and recovery phases and transmitted via Bluetooth to a compatible mobile device for data acquisition. The raw RR interval series will be exported for offline analysis. Prior to analysis, data will be visually inspected to identify artifacts, ectopic beats, and signal loss. Artifact correction and filtering procedures will be applied following established methodological recommendations for HRV analysis, and segments with excessive noise or non-physiological values will be excluded.

Time-domain HRV parameters will include the standard deviation of normal-to-normal intervals (SDNN), the root mean square of successive differences (RMSSD), and the percentage of successive NN intervals differing by more than 50 ms (pNN50). Frequency-domain analysis will be performed to obtain low-frequency (LF) and high-frequency (HF) components, expressed in absolute and normalized units, as well as the LF/HF ratio as an indicator of sympathovagal balance.

#### 3.2.3. Ergonomic Risk Analysis

Musculoskeletal risk will be evaluated using the Rapid Upper Limb Assessment (RULA) method [27], which assesses body posture, applied force, and repetitive movements. Scores will be assigned to head, trunk, upper limbs, wrists, and legs, as well as physical effort, allowing classification of ergonomic risk levels and the need for corrective interventions. The assessment will be conducted by the principal investigator, who has experience in ergonomic analysis, and will be performed during the ascent and descent tasks to capture posture and movement under real task conditions.

#### 3.2.4. Attention and Memory

Cognitive performance will be assessed using the NEUROPSI^®^ instrument (Pearson Assessments, San Antonio, TX, USA) [28], which evaluates attention, memory, executive function, and language through standardized tasks, including short-term memory, attentional capacity, visuospatial skills, and verbal fluency. The assessment will be analyzed by a trained professional with experience in neuropsychological testing under standardized conditions and will require approximately 15 min per participant. Participants experiencing adverse reactions during training will be withdrawn from the activity, and the event will be documented.

#### 3.2.5. Adverse Effects and Cybersickness Monitoring

To ensure participant safety, adverse effects associated with immersive virtual reality exposure, including nausea, dizziness, visual discomfort, disorientation, headache, and postural instability, will be actively monitored during and after the VR session. Participants will be instructed to report any discomfort immediately, and the session will be paused or discontinued if moderate or severe symptoms occur.

All adverse events will be documented, including type, severity, onset, and resolution. Participants presenting persistent or severe symptoms will be withdrawn from the intervention and referred for medical evaluation if necessary. Serious adverse events will be reported to the institutional ethics committee in accordance with regulatory requirements. The incidence and nature of adverse effects will be summarized and reported in the study results.

### 3.3. Outcomes

#### 3.3.1. Primary Outcome

The primary outcome will be overall performance in practical work-at-height skills, assessed immediately after completion of the practical training phase. Performance will be evaluated using a dichotomous pass/fail system across seven predefined domains: (1) correct donning and adjustment of the harness; (2) selection and installation of anchorage points compatible with the system and load requirements; (3) appropriate and continuous use of lifelines; (4) safe execution of ascent and descent while maintaining continuous connection; (5) proper securing of tools; (6) appropriate simulation of emergency response in accordance with established protocols; and (7) systematic inspection of personal protective equipment for fault identification.

Each domain will be assessed independently by certified instructors using a standardized checklist and will be classified as “pass” only when all predefined safety criteria for that domain are met. The composite primary outcome will be classified as “pass” only if the participant achieves a “pass” rating in all seven evaluated domains; failure in at least one domain will result in an overall classification of “fail.”

#### 3.3.2. Secondary Outcomes

Secondary outcomes include cardiac autonomic response assessed through HRV, ergonomic risk scores derived from RULA [27], and cognitive performance measured using NEUROPSI scores across attention, memory, executive function, and language domains.

### 3.4. Sample Size

The target population consists of approximately 400 individuals per year enrolling for the first time in authorized work-at-height training programs. As this study evaluates a device-based training intervention rather than a pharmacological treatment, it was designed as a pilot, hypothesis-generating trial aimed at estimating effectiveness, feasibility, and effect sizes to inform future large-scale studies, in accordance with methodological recommendations for early-phase device and training evaluations.

Sample size estimation was based on hypothesis testing for the comparison of two independent proportions, corresponding to the primary binary outcome (overall pass/fail performance in practical work-at-height skills). Assuming a pass rate of 60% in the control group and an expected improvement to 85% in the immersive virtual reality group, a total of 72 participants (36 per group) provides approximately 80% power to detect this 25% absolute difference at a two-sided α level of 0.05. To account for potential attrition and the operational challenges inherent to training settings, a final sample size of 78 participants was planned, equally allocated between groups (39 per group), with replacement of participants in cases of withdrawal to preserve statistical validity. Participant replacement in cases of withdrawal will be considered only during the recruitment phase to preserve group balance without introducing post-randomization bias.

Beyond statistical power considerations, the planned sample is consistent with established methodological guidance for pilot studies. Hertzog (2008) indicates that 30–40 participants per group are sufficient to estimate effect sizes with acceptable precision in hypothesis-generating research [29]. Furthermore, in the field of immersive virtual reality training, published studies frequently report smaller samples; our sample exceeds the median size observed in recent research, aligning with current practices for high-intensity technical interventions [30].

### 3.5. Randomization

Participants will be allocated to the intervention or control group using a block randomization strategy at the cohort level, based on weekly training groups scheduled by the National Learning Service of Colombia. Each training week constitutes a block, and blocks are assigned to either the control or intervention condition according to a predefined random allocation sequence generated prior to study initiation.

The allocation sequence is implemented by the institutional scheduling system and is not accessible to the research team, ensuring allocation concealment and minimizing investigator influence on group assignment. Due to the operational characteristics of the training program, individual-level randomization is not feasible; however, randomization at the block (cohort) level preserves the nature of group allocation. To address potential imbalances related to temporal clustering, baseline characteristics will be compared between groups, and adjusted analyses will be performed when appropriate.

### 3.6. Blinding

Outcome assessors and data analysts will be blinded to group allocation. Practical skills will be evaluated by certified instructors who will not be involved in the delivery of the training intervention and will be unaware of participants’ group assignment at the time of assessment. Due to the nature of the intervention, blinding of participants and training personnel is not feasible, as the use of immersive virtual reality is readily identifiable. To minimize potential bias, standardized assessment protocols and predefined objective criteria will be used across both groups. All datasets will be anonymized and coded prior to analysis to prevent identification of participant group assignment.

### 3.7. Data Collection and Management

Data will be collected in real time using coded identifiers to ensure confidentiality. Random audits will be conducted to verify consistency between raw equipment outputs, recorded forms, and database entries. Missing or inconsistent data will be addressed promptly, and participants with irretrievable missing data will be excluded from analysis.

### 3.8. Statistical Analysis

Statistical analyses will be conducted according to the prespecified primary and secondary outcomes. The primary outcome, defined as the composite pass/fail performance in practical work-at-height skills, will be analyzed as a binary variable. Between-group comparisons will be performed using the chi-square test or Fisher’s exact test, as appropriate, and effect sizes will be reported as risk ratios with 95% confidence intervals.

Secondary outcomes will be analyzed according to their measurement properties. Cardiac autonomic response, assessed through heart rate variability parameters, will be analyzed using repeated-measures mixed-effects models, with time and group as fixed effects and participant as a random effect, to account for within-subject correlations. When appropriate, post hoc comparisons will be adjusted using Bonferroni correction.

Ergonomic risk scores derived from the Rapid Upper Limb Assessment (RULA) and perceived workload measured using the NASA-TLX will be compared between groups using mixed-effects or repeated-measures models, depending on data structure. Cognitive performance outcomes will be analyzed descriptively and explored as potential predictors of training performance using correlation analyses and multivariable regression models. Assumptions of normality and homogeneity will be assessed prior to analysis. When appropriate, analysis of covariance (ANCOVA) or mixed-effects models adjusted for relevant covariates (e.g., age and sex) will be applied; training exposure (total time) will be included as a covariate when appropriate. Statistical significance will be set at *p* < 0.05.

### 3.9. Quality Assurance

An independent researcher will conduct periodic audits to verify data accuracy. Weekly reviews will be performed prior to database entry. Biosignal data will undergo filtering and noise reduction. Monthly research team meetings will monitor study progress.

## 4. Discussion and Expected Results

This research contributes to the advancement of the United Nations Sustainable Development Goals, particularly Goal 3 (Good Health and Well-being) and Goal 8 (Decent Work and Economic Growth). In the national context, these goals are especially relevant for promoting inclusive and sustainable economic development while simultaneously ensuring the protection of workers’ health and safety. Creating safe working environments is essential, as the economically active population represents a fundamental pillar of regional development.

In June 2022, the International Labour Organization formally recognized access to a safe and healthy working environment as a fundamental right [31]. Within this framework, the present study has the potential to generate a meaningful impact across several dimensions. By simulating hazardous occupational situations within controlled virtual environments, workers may acquire skills and knowledge that enhance their ability to identify and manage risks more effectively. This approach may contribute to increasing the proportion of hazards identified during real-world operations [32].

The use of immersive virtual reality may also reduce the costs and time associated with training for high-risk activities. This training modality enables the creation of accessible, repeatable, and flexible virtual environments, reducing the need for costly real-world training scenarios that require specialized equipment or complex logistics [33]. Furthermore, immersive virtual environments allow workers to practice decision-making in high-risk situations, which may improve the efficiency and effectiveness of job performance [34]. Additionally, the initial engagement associated with the novelty of immersive VR may transiently enhance motivation and learning; future studies should examine whether these effects persist once familiarity with the technology is established [30].

*Feasibility and Implementation Challenges in Local Contexts*

The practical feasibility of implementing immersive virtual reality at scale in real-world training centers should be considered, particularly in resource-constrained local contexts. Although standalone head-mounted displays such as the Meta Quest 2 reduce infrastructure needs by eliminating high-performance computers, with an approximate hardware cost of USD 250–370 per unit, several challenges remain. These include limited institutional budgets for initial procurement, device maintenance and replacement costs, connectivity constraints for content updates and multi-user environments, and the need for instructor training to ensure effective pedagogical integration. Once virtual environments are developed and validated, software costs can remain relatively low, about USD 300–1000 per year for institutional licensing or support, especially when platforms are designed and maintained locally; however, sustained technical support, periodic updates, and interoperability with existing training systems may pose additional barriers. Despite these challenges, local development may reduce long-term costs, enable adaptation to regional occupational needs, and strengthen technological capacity within training ecosystems.

The integration of emerging technologies into occupational training is additionally encouraged, promoting innovative and advanced solutions for worker safety and skill development. Collectively, these strategies have the potential not only to enhance safety and operational efficiency in work environments but also to strengthen organizational competitiveness at a regional level, while fostering safer and more productive workplaces [35].

*Linking Biosignals, Stress Management, and VR Training Outcomes*

From a physiological perspective, the analysis of biosignals during virtual reality-based training may provide a deeper understanding of human responses to stress and high-risk situations [36]. This information could facilitate the identification of specific biomarkers associated with stress or anxiety levels, thereby supporting the early identification of workers who may require additional training or support [37].

Finally, the findings of this research may contribute to the development of effective strategies for managing stress and anxiety in high-risk occupational settings, benefiting both workers and organizations. Moreover, by exploring the relationship between physiological responses and task performance, the study may help to further validate immersive virtual reality as a training tool. In addition, the three-month follow-up will provide preliminary insights into the durability of training effects and the retention of safety-related behaviors, contributing to the understanding of the long-term effectiveness of immersive virtual reality-based training. While the current sample size is statistically powered to detect the expected effects, we acknowledge that, as a pilot study, future multi-center trials with larger cohorts would further enhance the generalizability of these findings across diverse worker populations. Additionally, as this study includes only first-time trainees, the findings may not generalize to experienced workers; in such cases, immersive virtual reality may function better as a complementary or refresher training tool, depending on the context and learning objectives [38]. Future research should therefore evaluate its effectiveness in refresher training programs and in other high-risk occupational settings beyond construction, where task complexity, team coordination, and contextual hazards may influence training outcomes. Moreover, the preparatory VR session may introduce a training-dose effect or a time-on-task effect [39], a common limitation in VR-based training studies. Although focused on work-at-height activities, these advances may also have broader applicability in other domains, including sports training, healthcare education, and stress management contexts.

## 5. Conclusions

The results of this study are expected to provide evidence on the effectiveness of immersive virtual reality in enhancing work-at-height training by integrating practical, cognitive, ergonomic, and physiological outcomes. Immersive training may improve hazard recognition, decision-making, and skill acquisition while providing a safe environment for repeated practice. Biosignal measurements such as heart rate variability offer objective insight into workers’ stress and fatigue responses, supporting tailored training interventions. This research could inform future occupational safety strategies, reduce training costs, and contribute to safer and more efficient high-risk work environments.

## Data Availability

No datasets were generated or analyzed during the current study. All data relevant to this study will be made available upon completion of the study.

## Supplementary Materials

The following supporting information can be downloaded at https://www.mdpi.com/article/10.3390/mps9020055/s1. Figure S1: Study timeline and assessments. File S1: SPIRIT checklist for the study protocol. Reference [40] is cited in the Supplementary Materials.

## Abbreviations

The following abbreviations are used in this manuscript:

IVR	Immersive Virtual Reality
SENA	Servicio Nacional de Aprendizaje
HRV	Heart Rate Variability
RULA	Rapid Upper Limb Assessment
NASA-TLX	NASA Task Load Index
ILO	International Labour Organization
BIM	Building Information Modeling
