# Supplementary material for: Work at Heights Training: Conventional Approach with and Without Immersive Virtual Reality Study Protocol
Source: Methods Protoc. 2026 Apr 1;9(2):55. doi: 10.3390/mps9020055 (PMC13118859; doi:10.3390/mps9020055)
Supplement: Supplementary file 1 [file mps-09-00055-s001.zip › Figure S1. Study timeline and assessments.pdf]

Figure S1: CONSORT 2025 Flow Diagram

This figure represents the planned flow of participants; no outcome data are available at this stage.

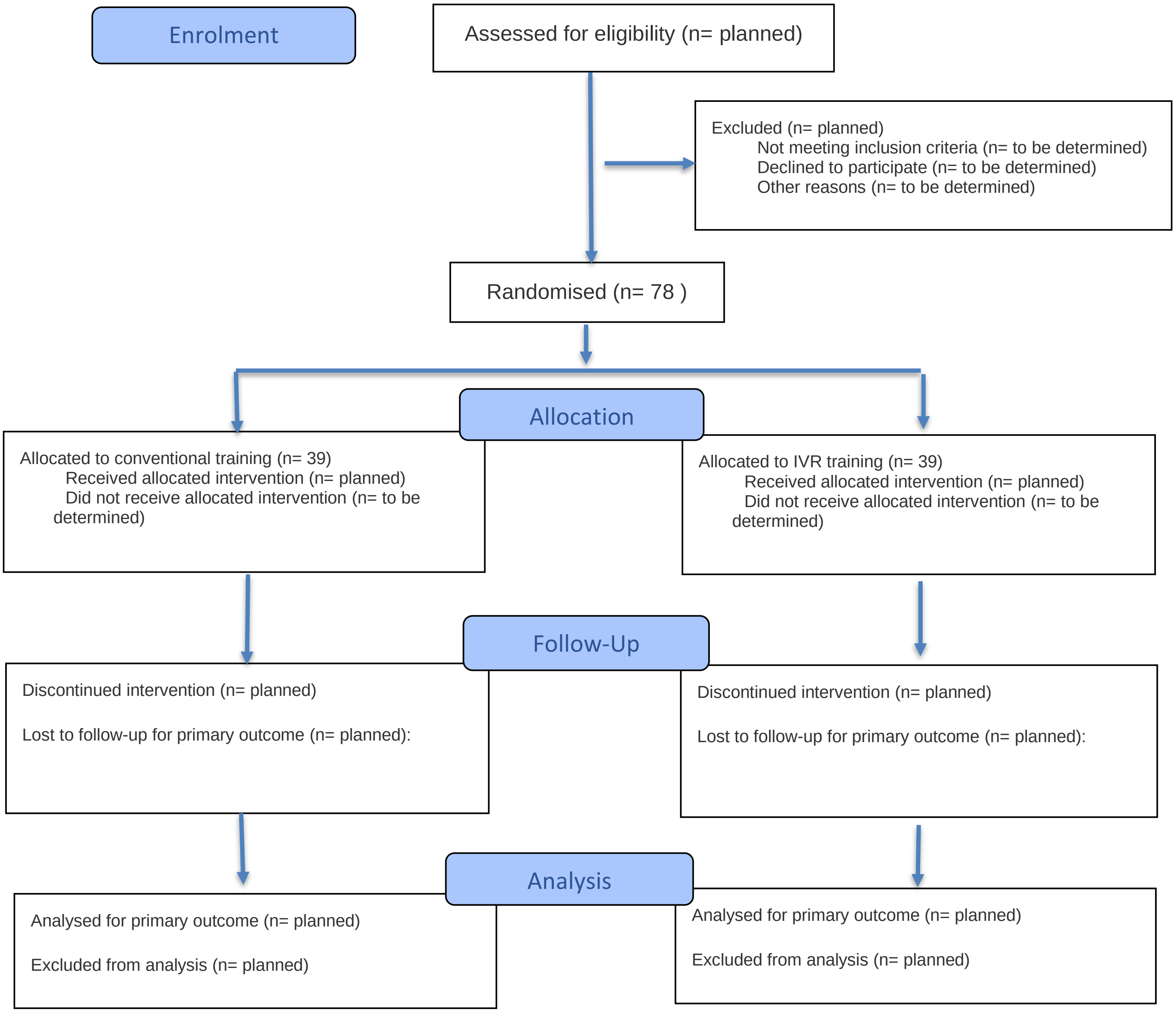

Citation: Hopewell S, Chan AW, Collins GS, Hróbjartsson A, Moher D, Schulz KF, et al. CONSORT 2025 Statement: updated guideline for reporting randomised trials. BMJ. 2025; 388:e081123. https://dx.doi.org/10.1136/bmj-2024-081123

© 2025 Hopewell et al. This is an Open Access article distributed under the terms of the Creative Commons Attribution License (https://creativecommons.org/licenses/by/4.0/), which permits unrestricted use, distribution, and reproduction in any medium, provided the original work is properly cited.
